# Association between SCN1A polymorphism rs3812718 and valproic acid resistance in epilepsy children: a case–control study and meta-analysis

**DOI:** 10.1042/BSR20181654

**Published:** 2018-12-18

**Authors:** Zhi Jian Wang, Jie Chen, Hai Liang Chen, Lin Yan Zhang, Duo Xu, Wen Ting Jiang

**Affiliations:** Department of Neurology, Fuzhou Neuropsychiatry Hospital, Fujian 350008, China

**Keywords:** Drug resistant, Epilepsy, Meta-analysis, Single Nucleotide Polymorphism, SCN1A, Valproic acid

## Abstract

Resistance to valproic acid (VPA), a first-line antiepileptic drug (AED), is occurring at an alarming rate, particularly in children. Signal nucleotide polymorphisms are considered crucial in this process. Therefore, we investigated whether the SCN1A polymorphism rs3812718 could be associated with VPA resistance. A total of 231 children with epilepsy who were solely administered VPA were enrolled. DNA was extracted from the peripheral blood samples and was genotyped by the Mass Array method. Furthermore, a meta-analysis was conducted between the drug responsive and resistant patients who were exposed to voltage-gated sodium channels. Results revealed that the TT genotype was associated with a higher risk of developing drug resistance (OR = 2.636, 95% CI 1.08–6.433, *P* = 0.033). After adjusting for the risk factors, a significant difference was still observed between the responsive and resistant groups (OR = 2.861, 95% CI 1.141–7.174, *P* = 0.025). Moreover, the recessive model was associated with a decreased drug resistance (OR = 0.402, 95% CI 0.167–0.968, *P* = 0.042) after correcting the risk factors. Meta-analysis of nine studies revealed similar results. In conclusion, our results proved that the rs3812718 TT genotype was associated with a high risk of developing drug resistance, and the recessive model could decrease the risk of VPA resistance.

## Introduction

Resistance to antiepileptic drugs (AEDs) is crucial in epilepsy treatment. About 50–60% patients can be effectively treated by a single AED, the remaining being resistant to the treatment [1]. Genetic polymorphisms are assumed to be a crucial source of interindividual differences in the pharmacokinetics of AED efficacy. Thus, evaluating the genetic factors that influence the pharmacokinetics of AEDs may help in improving individualized therapies [2].

Single nucleotide polymorphisms (SNPs) are reported to be associated with AED resistance, in particular, by blocking the voltage-gated sodium (Na^+^) channels [3,4]. The Na^+^ channel neuronal type I alpha subunit (SCN1A), which encodes the α-subunit of the Nav1.1 Na^+^ channel, is presumed to be associated with AED resistance. An SCN1A functional polymorphism (IVS5-91 rs3812718 C > T) located in the 5′ splice donor site was reported to generate alternative splicing products, resulting in altered proportions of exon 5′ transcripts in the neonate and adult brain tissue [5].

Existing evidence has demonstrated that the rs3812718 polymorphism can affect the carbamazepine (CBZ) and coadministered valproic acid (VPA) resistance epilepsy in a cross-sectional study of Japanese patients [6]. Tate et al. reported that patients with the SCN1A rs3812718 AA genotype showed reduced sensitivity to the Na^+^ channel-blocking drugs [7]. However, studies evaluating the genomic variants that influence the VPA dosage, specifically in children, are scarce [6,8].

VPA, a fatty acid with an anticonvulsant property for treating several epilepsies and seizures, is prescribed worldwide as an effective and economic first-line AED [2]. Nevertheless, it is reported that the mean efficacy of VPA ranged from 70% to 76% [9]. VPA blocks the voltage-gated Na^+^ channels; therefore, we speculated that the SCN1A genotype polymorphism may also be associated with a reduced therapeutic effect of VPA by altering the response of the Na^+^ channel [10]. The present study assessed the association between the rs3812718 polymorphism and VPA resistance in pediatric epilepsies, followed by a meta-analysis to evaluate the relationship of the polymorphism with AED resistance.

## Materials and methods

### Patients recruitment

Patients were consecutively enrolled between February and October 2017 from the outpatient and inpatient department of Fuzhou Neuropsychiatry Hospital. The inclusion criteria were: (1) patients aged 2–18 years with epilepsy [11]; (2) those receiving sustained-release tablets of VPA twice a day for at least 30 days; (3) those who received a VPA monotherapy; and (4) patients without other severe diseases. Exclusion criteria were: (1) patients with significant mental disorders; severe liver, renal, or cardiac impairment; and serious adverse drug reactions to VPA and (2) patients with Dravet or generalized epilepsy with febrile seizure plus. Informed consents were obtained from all patients or from their immediate relatives. The present study was approved by the Ethics Committee of Fuzhou Neuropsychiatry Hospital and all the researches were performed in accordance with the Declaration of Helsinki.

On enrollment, the patients provided their clinical history including the demographic data, dosage regimens, and seizure type and frequency (before drug intake and after 1-year of treatment). A 1-year follow-up was done to evaluate the therapeutic effect of VPA. Steady-state venous blood samples were collected after the regular intake of sustained-release VPA tablets twice a day for at least 30 days. VPA-resistant epilepsy was defined as a reduced percentage of seizure frequency (less than or equal to 50%) and reduced seizure frequency (more than 50% was regarded as responsive epilepsy) [12–14]. The seizures and epilepsy syndromes were classified according to the International League Against Epilepsy (ILAE) [15,16].

### Genotyping

Blood samples of all participants were collected and stored at −80°C until use. Genomic DNA was extracted from the whole blood using a commercial kit (Qiagen, Hilden, Germany) according to the manufacturer’s protocol. Patients’ DNA was purified by Qiagen DNA purification kit (Qiagen, Hilden, Germany). Genotyping was identified by MassArray method (Sequenom, USA).

### Meta-analysis

Studies published by 20 May, 2018 were identified by a systematic literature search in PubMed, Embase, Web of Knowledge, China National Knowledge Infrastructure (CNKI) and WanFang Data. MeSH and title/abstract were used for all eligible studies that mainly focus on the genotype of rs3812718 polymorphism either in VPA responsive or resistant patients. The search strategy was as follows: (‘seizure’ or ‘epilepsy’) AND (‘sodium channel neuronal type I alpha subunit’ or ‘SCN1A’ or ‘rs3812718’) AND (‘resistan*’ or ‘respons*’).

Eligible studies must met the following criteria: (1) the study evaluated the association between rs3812718 polymorphism and AED resistant, (2) the report focused on detailed genotype frequencies among human beings, and (3) the investigation was a case–control study. Accordingly, the exclusion criteria were as follows: (1) studies without detailed genotype data; and (2) reports with overlapping data. The ‘Search strategy’ was shown in Supplement Materials.

### Statistical analysis

All statistical analyses were performed by SPSS (version 17.0, SPSS Inc., Chicago, USA). Hardy–Weinberg equilibrium (HWE) was assessed by using a χ^2^ test and a *P* value <0.05 was considered significant disequilibrium. Differences in genotypic frequencies between groups were calculated by the Pearson’s chi-square test or Mann–Whitney *U* test. Odds ratios (ORs) with 95% CI were calculated. Logistic regression analysis was used to evaluate the contribution of genetic and non-genetic factors to the VPA resistant under dominant and recessive model. Patients’s age, body weight and seizure frequency were treated as risk factors to be adjusted in the analysis.

For meta-analysis, the OR and 95% confidence interval (95% CI) were calculated to assess the relationship of rs3812718 polymorphism between AEDs responsive and resistant. Pooled ORs were obtained from combination of single study under recessive model (TT vs. CT+CC) and dominant model (TT + CT vs. CC). Heterogeneity was evaluated by *Q* statistic and *I*^2^ statistic. Once, *Q*-test >0.10 or *I*^2^ < 50%, the fixed effect model (Mantel–Haenszelmethod) was used to calculate the pooled ORs, otherwise, the random-effect model (DerSimonian–Laird method) was used. The significance of the pooled ORs was assessed by *Z*-test, where *P* < 0.05 indicated statistically significant. Begg’s and Egger’s linear regression tests were applied to assess the potential publication bias. The statistical analyses were performed by using the STATA version 12.0 (STATA Corporation, College Station, TX, USA) [18].

## Results

### The characteristic of the study

A total of 231 (84 female) patients with a mean age of 9.80 ± 5.1 years and body weight 33.48 ± 16.2 kg were enrolled in the study. All patients were Chinese with confirmed epileptic diagnosis. Of these, 142 patients were classified as general type, 62 as focal type, and 27 as unable to be classified. The seizure frequency was less than once per day in 143 patients, between one and five times per day in 72 patients, and over five times per day in 16 patients. All participants were divided into two groups, VPA responsive (*n* = 185) and VPA resistant (*n* = 46). The demographics characteristics are listed in Table 1.

**Table 1:** The basic characteristic of the enrolled patients

Character	Total	Response	Resistant	*P*
*N* (female)	231 (94)	185 (76)	46 (18)	0.984
Age	9.80 ± 5.1	9.03 ± 4.08	7.52 ± 4.1	0.057
Body weight	33.48 + 16.2	34.36 + 15.43	29.74 + 13.76	0.054
Seizure type^a^	1.61 ± 4.45	1.4 ± 4.58	2.54 ± 3.77	0.855
1	142	117	25	
2	62	49	13	
3	27	19	8	
Frequency^b^				**0.001**
<1	143	125	18	
1–5	72	49	23	
>5	16	11	5.	
Genotype				**0.014**
CC	60	49	11	
CT	106	78	28	
TT	62	58	4	

a Seizure type: 1 represents general, 2 represents focal, and 3 represents unknown.

b Seizure frequency before treatment (times per day).

### Genetics association analysis

The genotypes of CC, CT, and TT were present in 25%, 48%, and 27% patients, respectively. The MAF (major allele frequency) of rs3812718 polymorphism was 0.4879. Deviation from the Hardy–Weinberg equilibrium was not observed (*P* > 0.05). The genotype distribution is listed in Table 2.

**Table 2:** The clinical parameters of all the patients according to SCN1A rs3812718 genotype

	CC	CT	TT	TT vs. CT + CC
				*P*
*N* (female)	60 (33)	106 (89)	65 (26)	0.338
Ages	8.39 ± 3.7	9.41 ± 4.18	8.09 ± 4.22	0.101
Body weight	31.72 ± 14.04	34 ± 15.54	30.47 ± 15.57	0.113
Frequency^a^	1.41 ± 2.79	2.08 ± 5.88	0.96 ± 2.01	0.484
<1	39	68	46	
1–5	17	39	16	
≥5	4	9	3	
Seizure type^b^				0.508
1	28	81	43	
2	21	285	16	
3	11	10	6	

a Seizure frequency before treatment (times per day).

b Seizure type: 1 represents general, 2 represents focal, and 3 represents unknown.

A genotype–phenotype correlation analysis between the SNP and VPA was performed. We found that the TT allele significantly correlated with the VPA resistant group (OR = 2.636, 95% CI 1.08–6.433, *P* = 0.033). Significant differences were still observed between the responsive and resistant groups (OR = 2.861, 95% CI 1.141–7.174, *P* = 0.025) even after adjusting the age, body weight, and seizure frequency (Table 3). In addition, the influence of genetic models (dominant and recessive model) on the VPA effect was also observed among the responsive and resistant groups. We did not find any significant associations in the recessive model (OR = 0.424, 95% CI 0.179–1.003, *P* = 0.051). However, after adjusting the age, body weight, and seizure frequency, a significant difference was observed between the two groups (OR = 0.402, 95% CI 0.16–0.968, *P* = 0.042; Table 4).

**Table 3:** The logistic regression analysis of rs3812718 SNP genotype with the drug resistant

rs3812718		OR	95% CI	*P*	OR^#^	95% CI^#^	*P*^#^
CC	Reference		1			1	
CT		1.86	0.67-5.164	0.234	1.927	0.687-5. 401	0.212
TT		2.636	1.08-6.433	**0.033**	2.861	1.141-7.174	**0.025**

Note: # were adjusted for age, body weight and seizure frequency. Significant associations are marked in bold.

**Table 4:** The logistic regression analysis of the dominant model and recessive model in VPA response group and resistant group

rs3812718	OR	95% CI	P	OR^#^	95% CI^#^	*P*^#^
Dominant model	1.068	0.504-2.262	0.864	1.067	0.499-2.281	0.867
Recessive model	0.424	0.179-1.003	0.051	0.402	0.167-0.968	**0.042**

Note: # were adjusted for age, body weight and seizure frequency. Significant associations are marked in bold.

Considering that the most significant association was observed with the recessive model, we further evaluated the relationship of genotype with VPA resistance by Poisson regression. The recessive model revealed a decreased risk of VPA resistance (OR = 0.688, 95% CI: 0.632–0.749). Meanwhile, the TT genotype with any clinical parameters was also analyzed, and no significant differences were observed between the patients carrying the TT genotype and those carrying the other two genotypes.

### Meta-analysis results

The study characteristics included in this meta-analysis are listed in Table 5. No significant publication bias was found in the Begg’s test (*P* > 0.05). A sensitivity analysis was conducted to assess the influence of each individual study on the pooled OR. The results revealed that no single study influenced the quality of the pooled ORs in the sensitivity analyses.

**Table 5:** Characteristics of individual studies included in the meta-analysis

Study	Ethnic	Age		Gender		Populations		Methods
		Responsive	Resistant	Responsive (M/F)	Resistant (M/F)	Drugs responsive	Drugs resistant	
Angelopoulou et al. [29]	Greece	40.76 ± 16.55	43.56 ± 16.37	62/68	37/33	130	70	TaqMan assay
Boting Zhou et al. [28]	Chinese	N/A	N/A	N/A	N/A	N/A	N/A	Illumina Sequencing
Luo Zhou et al. [30]	Chinese	22.36 ± 14.10	22.98 ± 11.21	144/91	103/53	235	156	Illumina Sequencing
Patrick Kwana et al. [31]	Chinese (Hongkong)	N/A	N/A	N/A	N/A	272	199	RT-PCR
Yuze Cao et al. [32]	Chinese	18.2 ± 16.8	19.0 ± 9.8	162	136	273	207	Illumina Sequencing
Blanca et al. [23]	Spainish	26.0 ± 19.8	27.0 ± 18.5	88/90	56/55	178	111	TaqMan assay
R. Kumari et al [33]	India	N/A	N/A	N/A	N/A	124	89	Real Time PCR
Ida Manna et al. [34]	Italy	N/A	N/A	N/A	N/A	401	482	Real Time PCR
Wang et al.	Chinese	9.13 + 4.01	7.79 + 4.01	144/85	38/22	229	60	MassArray
Haerian BS et al. [35]	Malay	28 ± 15	30 ± 15	70/56	64/61	126	125	MassArra
Haerian BS et al. [30]	India	30 ± 16	33 ± 17	49/43	38/32	92	70	MassArra
Haerian BS et al. [30]	Chinese	34 ± 19	33 ± 17	83/65	77/63	148	140	MassArra
Haerian BS et al. [30]	Chinese (HongKong)	38 ± 16	38 ± 14	180/156	225/242	336	467	MassArray

**Figure 1:**
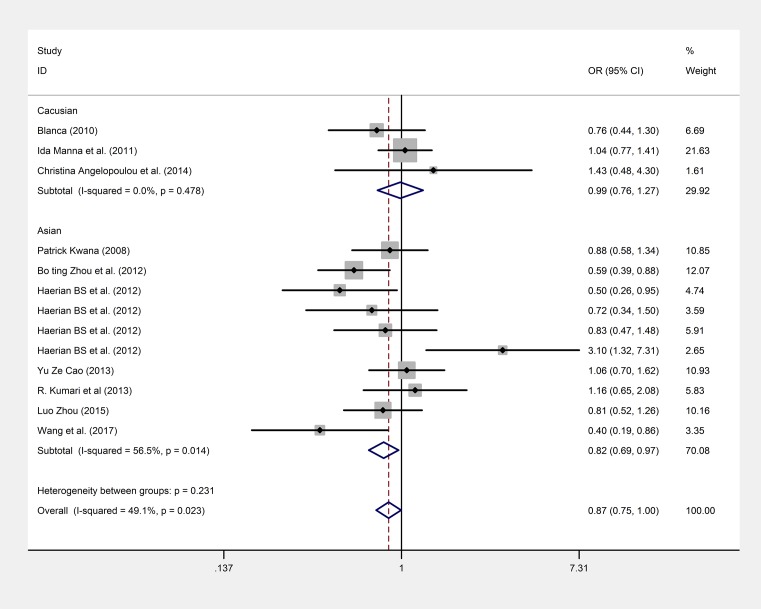
The forest plot of the SCN1A rs3812718 between drug resistant and responsive group under recessive model

**Figure 2:**
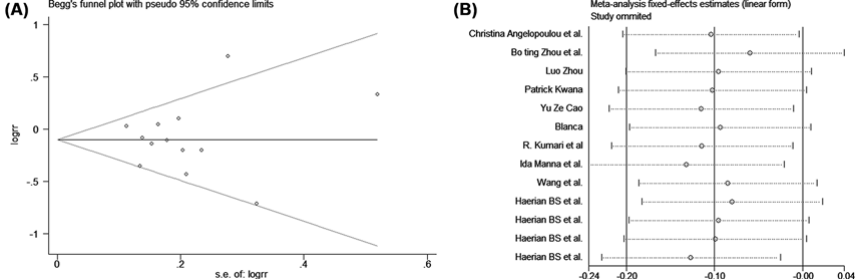
The evaluation of the publication bias under recessive model A is the Begg’s funner plot. B is the sensitivity analysis.

A pooled meta-analysis was also employed to observe the Na^+^ channel AEDS and its effect on epilepsy. Our results revealed that the recessive model can significantly decrease the risk of drug resistance (*P* = 0.037, OR = 0.9, 95% CI: 0.82–0.99) by using a fixed model. Subgroup analysis indicated the significant association of the rs3812718 polymorphism with drug resistance in the Asian population (*P* = 0.016, OR = 0.87, 95% CI: 0.78–0.98), but not in the Caucasian population (*P* = 0.906, OR = 0.99, 95% CI: 0.82–1.19; Figures 1 and 2). Nevertheless, we did not find any correlation between the rs3812718 polymorphism and drug resistance in other genetic models (dominant model, allelic model, heterozygous model, and homozygous model; the forest plot can be found in supplement materials).

## Discussion

Epilepsy is a common pediatric neurological disorder, with its prevalence ranging from 3000 to 6000 per million [19]. Administering AEDs is the core approach to control epileptic seizures; however, about 40% of patients still do not exhibit a satisfactory effect [20]. Approximately, 10–20% children develop drug-refractory epilepsy, with unclear etiologies [21]. Tate et al. reported that a common polymorphism in the SCN1A gene associates with the phenytoin serum levels during the maintenance dose [22]. Sánchez et al. reported that patients with symptomatic epilepsies with the ABCB1 3435 CT or TT genotypes revealed a lower risk of drug resistance than those with the CC genotype [23]. Despite discovering the majority of susceptibility polymorphism, the relation between the SNP and drug-resistant epilepsy remains unclear.

Drugs affecting the voltage-gated Na^+^ channels were most frequently used in treating epilepsy. VPA, with a mechanism of blocking the Na^+^ channel, remains the first choice for children with focal or generalized epilepsy. Although it can be used for treating several epilepsies and seizures, its association with the SCN1A rs3812718 polymorphism remained unclear. In the present study, we found that patients with the TT genotype easily developed drug resistance. Using the recessive model (TT vs. CT + CC), we found a significant correlation between the maximum doses of AEDs and carriers of the C allele (C/T or C/C genotype). Our results were partially consistent with the findings of Kazuko Nakagawa et al. [6], which were further proved by our meta-analysis results from 9 relative studies.

VPA emerges with a broad-spectrum efficacy range, which considerably varies among patients consuming the same dose of VPA. Although VPA therapy is relatively safe, epilepsy control with VPA can be a difficult and lengthy process owing to the drug’s narrow therapeutic index [24]. In addition, adverse drug reactions, particularly liver toxicity in young children, are also relatively common and often require dose adjustment [25]. Gene polymorphisms such as rs3812718 G > A may be related to toxic reactions and idiosyncratic adverse effects, which may explain the underlying mechanisms and possible prevention of drug resistance.

Mutations in Na^+^ channel transcripts in human epilepsy have been discovered in the brain tissue. Genetic variants from ion channels and functionally related genes may affect the response to first-line AEDs; thus, identifying genetic markers in the treatment response has been an urgent need in epilepsy therapeutics [26,27]. SCN1A rs3812718 polymorphism has been attributed to be a possible modifying factor for epilepsy therapeutics, particularly in the Asian populations. Zhou et al. reported that the polymorphism is significantly associated with the retention rate of CBZ monotherapy in a Chinese Han population [28]. Nevertheless, results from the replication studies have been inconsistent, with few negative results as well. Sánchez et al. reported no significant correlations between the SCN1A rs3812718 polymorphism and drug resistance in a Caucasian population [23].

This is the first study to report the significant association of SCN1A polymorphism (IVS5-91 rs3812718 C > T) with VPA resistance among children. Although our results are promising and may help in identifying patients with VPA resistance, these should be considered as preliminary results and further research is necessary. Moreover, the present study has certain limitations. First, the considerably complex mechanism of VPA and our focus solely on the Na^+^ channels fails to efficiently explain the drug resistance of VPA. Second, as the sample size was small, few indices in our research were nearly at the edge of the significance. Third, as fewer studies were available on the relationship between rs3812718 polymorphism and VPA resistance, we had to consider all the published articles that evaluated the effects of AEDs on the Na^+^ channel, eventually leading to diverse analyses.

In conclusion, our study had for the first time showed an association between the resistant and responsive of VPA and rs3812718 polymorphism. However, these data need to be replicated in a larger cohort of patients, and functional studies will be necessary to investigate whether and how rs3812718 polymorphism might influence key pathways involved in the pathogenesis of epilepsy and seizure.

## Abbreviations

AED: antiepileptic drug
CBZ: carbamazepine
MAF: major allele frequency
OR: odds ratio
SNP: single nucleotide polymorphism
VPA: valproic acid
